# Serum thiols and cardiovascular risk scores: a combined assessment of transsulfuration pathway components and substrate/product ratios

**DOI:** 10.1186/1479-5876-11-99

**Published:** 2013-04-15

**Authors:** Arduino A Mangoni, Angelo Zinellu, Ciriaco Carru, John R Attia, Marc McEvoy

**Affiliations:** 1 Division of Applied Medicine, Section of Translational Medical Sciences, School of Medicine and Dentistry, University of Aberdeen, Polwarth Building, Foresterhill, Aberdeen AB25 2ZD, UK; 2 Department of Biomedical Sciences, University of Sassari, Viale San Pietro 43/b, Sassari 07100, Italy; 3 Centre for Clinical Epidemiology & Biostatistics, Hunter Medical Research Institute, School of Medicine and Public Health, University of Newcastle, Callaghan, NSW 2308, Australia

**Keywords:** Thiols, Transsulfuration, Cardiovascular risk, Epidemiology, risk assessment

## Abstract

**Background:**

Serum thiols have shown associations with surrogate markers of cardiovascular disease. However, little information is available on their combined association with validated cardiovascular risk scores for primary prevention at population level. We sought to determine whether individual serum thiol concentrations and substrate/product ratios within the transsulfuration pathway are independently associated with such scores.

**Methods:**

Data on clinical and demographic characteristics, serum thiols (homocysteine, cysteine, taurine, glutamylcysteine, total glutathione and cysteinylglycine) and high-sensitivity C-reactive protein (CRP) were collected from a sample of the Hunter Community Study without previous cardiovascular events [n=350, median age (IQR) = 62 (59–66) years]. Five-year absolute cardiovascular risk score for each subject was calculated using the Framingham Risk Equation.

**Results:**

Median risk score was 7% (IQR 4–10). After adjusting for body mass index, estimated glomerular filtration rate and physical activity regression analysis showed independent associations between cardiovascular risk scores and a) higher serum homocysteine (B 0.066, 95% CI 0.040 to 0.091, *P*<0.001) and lower cysteine (B −0.003, 95% CI −0.005 to −0.001, *P*=0.003) and glutathione (B −0.029, 95% CI −0.056 to −0.003, *P*=0.03) concentrations; and b) higher homocysteine/cysteine (B 0.114, 95% CI 0.066 to 0.161, *P*<0.001) and lower glutathione/cysteinylglycine (B −1.145, 95% CI −2.030 to −0.260, *P*=0.011) ratios. No significant associations were observed between cardiovascular risk scores, taurine and CRP.

**Conclusions:**

Serum homocysteine, cysteine and glutathione are independently associated with cardiovascular risk scores at population level. Enzymatic pathways involved in reduced bioconversion of homocysteine into cysteine and increased glutathione degradation might play an important role in such associations.

## Background

Despite significant advances in the understanding of the processes underlying the onset and progression of atherosclerosis and thrombosis a significant proportion of cardiovascular events cannot be predicted with the available risk factors. This suggests that additional pathophysiological mechanisms are involved. Potential biomarkers of cardiovascular risk should be measurable in the population. Moreover, they should be significantly related with cardiovascular risk and modifiable by means of pharmacological and/or non-pharmacological interventions [1].

**Figure 1:**
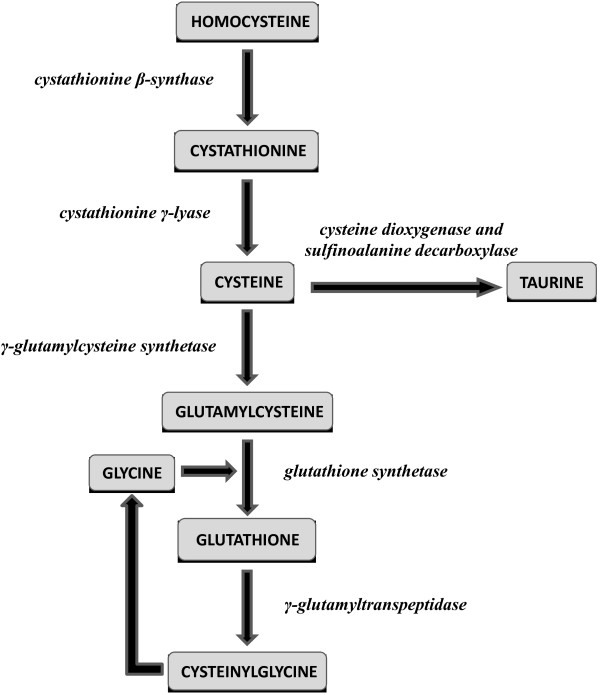
The transsulfuration pathway.

The highly reactive sulphur-containing amino acid homocysteine has long been shown to exert detrimental effects on vascular homeostasis by inhibiting nitric oxide synthesis and promoting oxidative stress and inflammation [2]. Several studies have shown that higher plasma/serum homocysteine concentrations independently predict adverse cardiovascular outcomes and improve risk reclassification [2,3]. Homocysteine is the initial step of the transsulfuration pathway [4]. The latter plays a crucial role, not only for homocysteine clearance but also for the synthesis of important homeostatic thiols such as cysteine, taurine and the natural antioxidant glutathione (Figure 1) [4].

Several studies have reported associations between plasma/serum transsulfuration pathway thiol concentrations and surrogate markers of cardiovascular disease, e.g. endothelium-dependent vasodilatation, intima-media thickness, and plaque burden [5-7]. Homocysteine, cysteine and glutathione also play an important role in modulating specific atherosclerotic processes, e.g. oxidation of low-density lipoproteins (LDL) and inflammation [8,9]. However, little knowledge is currently available on whether there are independent associations between individual transsulfuration pathway components and robust measures of cardiovascular risk for primary prevention, calculated using validated scoring systems at population level. Ideally, human studies investigating such associations should further account for clinical confounders affecting both thiol concentrations and cardiovascular risk [10,11]. The identification of associations with scores directly predictive of cardiovascular risk would further enhance the clinical relevance, and potential use, of thiols as biomarkers. We addressed this issue by examining the combined associations between transsulfuration pathway thiols and absolute cardiovascular risk scores at population level, in an established epidemiological cohort of human ageing. Associations between thiols and cardiovascular risk scores were evaluated looking both at individual thiols and at substrate/product ratios within the transsulfuration pathway. Substrate/product ratios were considered markers of the activity of specific enzymes in the pathway (Figure 1). Analyses were further adjusted for a recently proposed cardiovascular risk marker, C-reactive protein, as well as body mass index, estimated glomerular filtration rate and mean daily step count, a marker of physical activity.

## Methods

### Population

The Hunter Community Study (HCS), a collaboration between the University of Newcastle’s School of Medicine and Public Health and the Hunter New England Area Health Service, is a population-based cohort study on human ageing [12]. Participants, a cohort of community-dwelling subjects aged 55–85 years residing in Newcastle (New South Wales, Australia), were randomly selected from the electoral roll and contacted between December 2004 and December 2007. Invitation letters were sent to 9,784 individuals. Of the 7,575 subjects for whom a response was received, 258 were ineligible (148 did not speak English, 92 were deceased, and 18 had moved to an aged-care facility), 3,440 refused and 3,877 initially agreed to participate. Of these, a total of 3,253 actually participated (response rate 44.5%).

After informed, written consent was obtained, subjects were asked to complete two self-report questionnaires and to return these when they attended the HCS data collection centre, during which time a series of clinical and biochemical measures was obtained. Clinical assessment included a full physical examination and measurement of blood pressure, heart rate, body mass index and waist-to-hip ratio. Routine haematological and biochemical parameters included full blood count, high-sensitivity C-reactive protein (CRP), fasting lipids, liver and renal function and fasting blood glucose. Additional samples were cryopreserved at −86°C and −196°C. Consent to link personal information obtained during the study to data from Medicare Australia and local health databases was also sought.

After the clinical assessment a further package of three self-reporting questionnaires to be returned by reply-paid post was given to participants to complete at home. The questionnaires provided details on demographic and socioeconomic characteristics, nutritional assessment, medical and surgical history, medication exposure, tobacco use and alcohol consumption [12].

### Absolute cardiovascular risk

The absolute cardiovascular risk score was calculated in each study subject using the Framingham Risk Equation [13]. The use of this cardiovascular scoring system has been advocated by the National Vascular Disease Prevention Alliance as a tool to quantify and to reduce the cardiovascular disease burden in the Australian population [14]. The equation allows calculation of a risk score that can then be classified as low (<10%), moderate (10-15%) or high (>15%) risk of a cardiovascular event, i.e. myocardial infarction, stroke or peripheral arterial occlusion, within five years in subjects without previous cardiovascular events and includes the following parameters: age (35–74 years), gender, systolic blood pressure (average of two office blood pressure measurements), total and HDL cholesterol, smoking, diabetes, and electrocardiographic parameters of left ventricular hypertrophy [13]. The following patient categories were automatically considered at increased risk of cardiovascular disease and did not allow specific score calculations: diabetes and age >60 years, a previous diagnosis of familial hypercholesterolaemia, systolic blood pressure ≥180 mmHg and total cholesterol >7.5 mmol/L [13].

The sample for this investigation (n=500) was derived from the initial cohort by simple random sampling. Of the 500 subjects, 150 were removed from the final analysis for the following reasons: previous cardiovascular events, missing thiol data, age >74 years and those automatically considered at increased risk of cardiovascular disease. Therefore, complete data were available for 350 subjects. A comparison of this sample with the entire cohort showed no significant difference for a range of clinical, biochemical, socioeconomic, and behavioural factors (data not shown). The HCS was performed according to the Declaration of Helsinki. All procedures were approved by the local ethics committee.

### Biochemical measurements

Blood was collected in EDTA tubes and centrifuged at 4°C and 3,000g for 10 min to separate plasma, which was stored for three years at −80°C before analysis. Serum concentrations of the thiols homocysteine, cysteine, cysteinylglycine, glutamylcysteine, glutathione and taurine were measured by laser-induced fluorescence capillary electrophoresis on 0.05 mL serum for taurine and 0.2 mL for the other thiols [15,16]. A five-point calibration curve was used to measure analyte concentrations. Only for taurine was homocysteic acid used as internal standard [16]. The minimum detectable concentration for all analytes was between 200 and 300 pmol/L, with mean recovery between 98% and 102%. A good reproducibility of intra-assay (CV <3.5%) and inter-assay (CV <6.4%) tests was obtained [15,16].

In addition to individual thiols, the following substrate/product ratios were calculated as markers of enzymatic activity along the transsulfuration pathway: homocysteine/cysteine (cystathionine β-synthase and cystathionine γ-lyase), cysteine/taurine (cysteine dioxygenase and sulfinoalanine decarboxylase), cysteine/glutamylcysteine (gamma-glutamylcysteine synthetase), glutamylcysteine/glutathione (glutathione synthethase), and glutathione/cysteinylglycine (γ-glutamyltranspeptidase) (Figure 1) [4].

High-sensitivity CRP was measured in serum by latex-enhanced immunoturbidimetry [17]. Estimated glomerular filtration rate (eGFR) was calculated using the Modification of Diet in Renal Disease formula [18].

### Physical activity

Step counts were obtained using a Yamax Digiwalker SW-200 pedometer (Yamasa Tokei Keiki Co Ltd, Tokyo, Japan) worn by participants for seven days. The mean daily step count, a marker of physical activity, was used as a continuous variable in the analysis [19,20].

### Statistical analysis

Results are expressed as means ± SD, medians and interquartile ranges, or frequencies as appropriate. Variables were tested for normal distribution by using the Kolmogorov-Smirnov test. Univariate associations between clinical variables, thiol concentrations, CRP and cardiovascular risk scores were assessed by Spearman’s rank correlation coefficient. The following variables, identified *a priori* to be associated with cardiovascular risk, were entered into forward stepwise linear regression analysis to identify independent associations with actual cardiovascular risk scores: body mass index, eGFR, daily number of steps, CRP and serum thiol concentrations (model a). Further analyses included the substrate/product ratios homocysteine/cysteine, cysteine/taurine, cysteine/glutamylcysteine, glutamylcysteine/glutathione and glutathione/cysteinylglycine (model b). We confirmed that the assumptions of linearity, normal distribution and equal variance were met. Multicollinearity was tested by measuring the tolerance and the variance inflation factor values for each analysis.

Although no *a priori* sample size was determined, assuming that at least 10–15 subjects are needed for each independent variable included in the multivariate analysis the sample size (n=350) was more than sufficient to accommodate the number of co-variables examined in this investigation (model a: ten variables; model b: eight variables). Analyses were performed using IBM SPSS Statistics 19.0 for Windows (SPSS Inc, Chicago, IL, USA). A two-sided *P*<0.05 indicated statistical significance.

## Results

**Figure 2:**
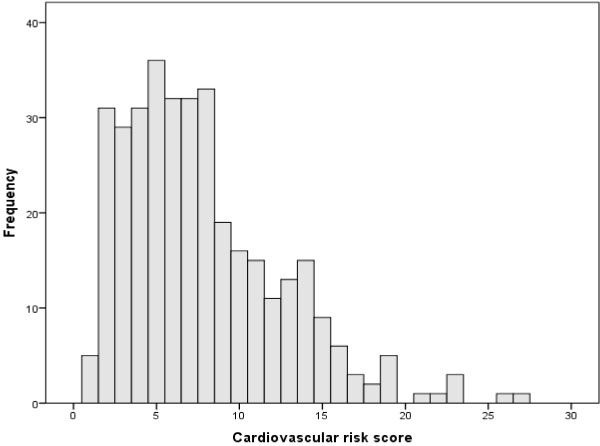
Distribution of the cardiovascular risk scores.

Clinical, demographic and biochemical characteristics of the study population are described in Table 1. The distribution of the cardiovascular risk scores was markedly skewed towards the left (Figure 2). Therefore, log transformed cardiovascular risk scores were entered into linear regression analysis.

**Table 1:** Clinical, demographic and biochemical characteristics

**Variable**	**Study population (n=350)**
Age [years, median (IQR)]	62 (59–66)
Females (%)	52.6
Current smoker (%)	7.7
Current alcohol use (%)	72.2
Body mass index [Kg/m^2^, median (IQR)]	28.0 (25.7-31.2)
Daily number of steps [median (IQR)]	6,903 (4,718-8,824)
Systolic blood pressure (mmHg, mean±SD)	135±16
Diastolic blood pressure (mmHg, mean±SD)	80±10
Heart rate (b/min, mean±SD)	66±10
Hypertension (%)	43.8
Rheumatoid arthritis (%)	5.2
Diabetes (%)	1.7
Hypercholesterolaemia (%)	36.7
Cardiovascular risk score [%, median (IQR)]	7 (4–10)
Low (%)	70.9
Moderate (%)	22.5
High (%)	6.6
Non steroidal anti-inflammatory drugs (%)	8.9
Beta-blockers (%)	17.0
Angiotensin converting enzyme inhibitors (%)	42.2
Calcium-channel blockers (%)	9.6
Statins (%)	28.1
Diuretics (%)	10.0
Antidiabetic drugs (%)	1.5
Folic acid supplements (%)	1.4
Fasting serum glucose [mmol/L, median (IQR)]	4.8 (4.4-5.2)
Total cholesterol (mmol/L, mean±SD)	5.2±0.9
LDL-cholesterol (mmol/L, mean±SD)	3.2±0.8
HDL-cholesterol [mmol/L, median (IQR)]	1.3 (1.1-1.5)
Triglycerides [mmol/L, median (IQR)]	1.1 (0.8-1.6)
eGFR^a^ (mL/min, mean±SD)	81±16
C-reactive protein [mg/L, median (IQR)]	1.9 (1.2-3.5)
Homocysteine [μmol/L, median (IQR)]	8.8 (7.6-10.5)
Cysteine (μmol/L, mean±SD)	183.1±36.5
Taurine [μmol/L, median (IQR)]	62.0 (51.6-83.6)
Glutamylcysteine (μmol/L, mean±SD)	4.2±1.1
Glutathione [μmol/L, median (IQR)]	3.9 (3.0-5.2)
Cysteinylglycine [μmol/L, median (IQR)]	28.6 (25.2-32.9)

a: calculated using the Modification of Diet in Renal Disease formula.

### Univariate associations

There were significant positive correlations between serum homocysteine and cysteinylglycine concentrations and cardiovascular risk scores (Table 2). A higher body mass index was significantly associated with higher cysteine and lower glutathione concentrations, lower eGFR, lower number of daily steps, higher CRP concentrations and higher cardiovascular risk scores. A lower eGFR was significantly associated with higher concentrations of all thiols, except for glutathione, higher body mass index and lower number of daily steps. Furthermore, there were significant correlations between a lower number of daily steps and higher homocysteine, cysteine and glutamylcysteine concentrations, higher body mass index, reduced eGFR and higher cardiovascular risk scores. CRP concentrations did not show any significant associations apart from a positive association with body mass index (Table 2).

**Table 2:** Univariate associations between body mass index, eGFR, daily steps, thiols, C-reactive protein, and cardiovascular risk scores

	**BMI**	**eGFR**	**Steps**	**Hcy**	**Cys**	**Tau**	**Glucys**	**GSH**	**Cysgly**	**CRP**	**CV score**
**BMI**	-	r= −0.01	r= −0.30	r= +0.10	r= +0.19	r= +0.03	r= +0.04	r= −0.17	r= −0.03	r= +0.32	r= +0.12
		*P*=0.05	*P*<0.001	*P*=0.07	*P*=0.001	*P*=0.60	*P*= 0.49	*P*= 0.002	*P*=0.60	*P*<0.001	*P*=0.02
**eGFR**	r= −0.01	-	r= +0.15	r= −0.34	r= −0.29	r= −0.16	r= −0.22	r= −0.06	r= −0.22	r= −0.01	r= −0.09
	*P*=0.05		*P*=0.005	*P*<0.001	*P*<0.001	*P*=0.004	*P*<0.001	*P*=0.27	*P*<0.001	*P*=0.82	*P*=0.11
**Steps**	r= −0.30	r= +0.15	-	r= −0.13	r= −0.13	r= 0.02	r= −0.16	r=0.03	r=0.04	r= −0.10	r= −0.24
	*P*<0.001	*P*=0.005		*P*=0.01	*P*=0.02	*P*=0.68	*P*=0.005	*P*=0.59	*P*=0.48	*P*=0.06	*P*<0.001
**Hcy**	r= +0.10	r= −0.34	r= −0.13	-	r= +0.53	r= +0.05	r= +0.07	r= −0.02	r= +0.37	r= −0.02	r= +0.19
	*P*=0.07	*P*<0.001	*P*=0.01		*P*<0.001	*P*=0.33	*P*=0.19	*P*=0.78	*P*<0.001	*P*=0.74	*P*<0.001
**Cys**	r= +0.19	r= −0.29	r= −0.13	r= +0.53	-	r= +0.03	r= +0.28	r= +0.11	r= +0.28	r= +0.01	r= −0.01
	*P*=0.001	*P*<0.001	*P*=0.02	*P*<0.001		*P*=0.54	*P*<0.001	*P*=0.06	*P*<0.001	*P*=0.79	*P*=0.79
**Tau**	r= +0.03	r= −0.16	r= 0.02	r= +0.05	r= +0.03	-	r= +0.11	r= +0.25	r= +0.28	r= +0.07	r= −0.01
	*P*=0.60	*P*=0.004	*P*=0.68	*P*=0.33	*P*=0.54		*P*=0.05	*P*<0.001	*P*<0.001	*P*=0.24	*P*=0.88
**Glucys**	r= +0.04	r= −0.22	r= −0.16	r= +0.07	r= +0.28	r= +0.11	-	r= +0.42	r= +0.22	r= +0.02	r= +0.01
	*P*= 0.49	*P*<0.001	*P*=0.005	*P*=0.19	*P*<0.001	*P*=0.05		*P*<0.001	*P*<0.001	*P*=0.65	*P*=0.92
**GSH**	r= −0.17	r= −0.06	r=0.03	r= −0.02	r= +0.11	r= +0.25	r= +0.42	-	r= +0.32	r= −0.07	r= −0.85
	*P*= 0.002	*P*=0.27	*P*=0.59	*P*=0.78	*P*=0.06	*P*<0.001	*P*<0.001		*P*<0.001	*P*=0.21	*P*=0.13
**Cysgly**	r= −0.03	r= −0.22	r=0.04	r= +0.37	r= +0.28	r= +0.28	r= +0.22	r= +0.32	-	r= +0.03	r= +0.13
	*P*=0.60	*P*<0.001	*P*=0.48	*P*<0.001	*P*<0.001	*P*<0.001	*P*<0.001	*P*<0.001		*P*=0.54	*P*=0.02
**CRP**	r= +0.32	r= −0.01	r= −0.10	r= −0.02	r= +0.01	r= +0.07	r= +0.02	r= −0.07	r= +0.03	-	r= +0.01
	*P*<0.001	*P*=0.82	*P*=0.06	*P*=0.74	*P*=0.79	*P*=0.24	*P*=0.65	*P*=0.21	*P*=0.54		*P*=0.87
**CV score**	r= +0.12	r= −0.09	r= −0.24	r= +0.19	r= −0.01	r= −0.01	r= +0.01	r= −0.85	r= +0.13	r= +0.01	-
	*P*=0.02	*P*=0.11	*P*<0.001	*P*<0.001	*P*=0.79	*P*=0.88	*P*=0.92	*P*=0.13	*P*=0.02	*P*=0.87	

BMI: body mass index; eGFR: estimated glomerular filtration rate; Hcy: homocysteine; Cys: cysteine; Tau: taurine; Glucys: glutamylcysteine; GSH: glutathione; Cysgly: cysteinylglycine; CRP: C-reactive protein; CV score: cardiovascular risk score.

### Regression analysis

After correcting for body mass index, eGFR, number of daily steps and CRP, regression analysis showed that higher cardiovascular risk scores were independently associated with higher concentrations of homocysteine and lower concentrations of cysteine and glutathione (model a, Table 3). Furthermore, analysis of substrate/product ratios showed that higher homocysteine/cysteine and lower glutathione/cysteinylglycine ratios were both independently associated with higher cardiovascular risk scores (model b, Table 4). No independent associations were observed between CRP concentrations and cardiovascular risk scores.

**Table 3:** Regression of cardiovascular risk scores model (a)

**Variables**	**B coefficient (95% CI)**	***P*-value**
(Constant)	2.380 (2.036 to 2.723)	<0.00001
Number of daily steps*	−0.004 (−0.006 to −0.002)	0.00003
Homocysteine	0.066 (0.040 to 0.091)	<0.00001
Cysteine	−0.003 (−0.005 to −0.001)	0.003
Glutathione	−0.029 (−0.056 to −0.003)	0.03

Variables entered in the model: body mass index, number of daily steps, eGFR, C-reactive protein, homocysteine, cysteine, taurine, glutamylcysteine, glutathione, cysteinylglycine, *: divided by 100.

**Table 4:** Regression of cardiovascular risk scores model (b)

**Variables**	**B coefficient (95% CI)**	***P*-value**
(Constant)	1.926 (1.598 to 2.255)	<0.00001
Number of daily steps*	−0.004 (−0.006 to −0.002)	0.00001
Homocysteine/cysteine#	0.114 (0.066 to 0.161)	<0.00001
Glutathione/cysteinylglycine	−1.145 (−2.030 to −0.260)	0.011

Variables entered in the model: body mass index, number of daily steps, eGFR, C-reactive protein, homocysteine/cysteine, cysteine/taurine, cysteine/glutamylcysteine, glutamylcysteine/glutathione, glutathione/cysteinylglycine.

*: divided by 100; #: multiplied by 100.

Five study subjects (1.4%) were on supplements containing folic acid at the time of assessment (Table 1). After adjusting for age, gender, body mass index, eGFR and number of daily steps folic acid intake was not significantly associated with any of the serum thiol concentrations (data not shown).

## Discussion

Our study showed significant independent associations between several transsulfuration pathway thiols and five-year cardiovascular risk, measured using the Framingham Risk Equation for primary prevention, in an established cohort of human aging. Serum concentrations of homocysteine showed positive associations with risk scores whereas cysteine and glutathione were negatively associated. Further assessment of substrate/product ratios within the transsulfuration pathway suggests that a reduced biotransformation of homocysteine into cysteine and an increased degradation of glutathione into cysteinylglycine are independently associated with higher cardiovascular risk scores and might play an important role in this context. Associations between thiols, substrate/product ratios and cardiovascular risk scores were independent of important clinical confounders, e.g. body mass index, renal function, physical activity and C-reactive protein, another cardiovascular biomarker.

The independent associations between higher homocysteine concentrations and cardiovascular risk support the existing evidence on the detrimental effects of this thiol on endothelial function and vascular homeostasis [2]. A recent study has further highlighted the role of homocysteine as independent predictor of cardiovascular events as well as a tool to improve risk classification [3]. Homocysteine lowering treatment, e.g. B-vitamin supplementation with folic acid, has been proposed as a safe and relatively inexpensive strategy to reduce cardiovascular risk [2]. However, a number of randomised placebo-controlled trials have failed to show any significant effect of homocysteine lowering on cardiovascular morbidity and mortality in different patient groups [21]. Notably, these studies were conducted in patients with pre-existing cardiovascular disease [21]. It has been suggested that patients in these studies had fairly advanced atherosclerotic disease associated with significant vascular structural and functional abnormalities, largely irreversible despite homocysteine lowering [22]. Moreover, concomitant treatment with other drugs for secondary prevention, e.g. aspirin and statins, might have masked the potential beneficial effects of homocysteine lowering treatment, reducing the possibility of observing a difference in cardiovascular outcomes between the active and placebo groups [23,24]. It remains largely unknown whether homocysteine lowering interventions conducted at an earlier stage, as part of a primary prevention strategy, might yield a different result [24]. The strong association between serum homocysteine concentrations and cardiovascular risk scores in patients without previous cardiovascular events in our study should prompt further intervention trials in similar populations. However, our study also suggests that other thiols within the transsulfuration pathway might play a role in modulating cardiovascular risk.

Higher serum cysteine concentrations have shown significant associations with surrogate markers of vascular disease, e.g. reduced endothelium-dependent vasodilatation [6]. By contrast, other studies have failed to demonstrate a significant role of cysteine in the pathophysiology of atherosclerosis after adjusting for several clinical and demographic confounders [5,25]. Moreover, no associations between cysteine concentrations and cardiovascular mortality were observed in a longitudinal study [26]. The contrasting results of these studies suggest the presence of a complex relationship between cysteine, atherosclerosis and cardiovascular disease. At least two factors might explain such relationship. First, a number of studies have reported significant associations between higher cysteine concentrations, higher body mass index and reduced glomerular filtration rate [10,11]. Univariate analyses in our study showed no associations between cysteine and cardiovascular risk scores. However, there were significant associations between cysteine, body mass index and glomerular filtration rate, in line with the available evidence [10,11]. This suggests that further studies on the potential role of cysteine in modulating cardiovascular risk should account for these clinical confounders in regression analysis. Second, the effects of cysteine on atherosclerosis might be concentration-dependent. A study has demonstrated that cysteine modulates the copper and iron-dependent oxidation of low-density lipoproteins (LDL), an important step in atherogenesis [8,27]. Cysteine concentrations ≥50 μmol/L fully prevented copper-dependent LDL oxidation. By contrast, concentrations between 25 and 1,000 μmol/L facilitated iron-dependent LDL oxidation in a concentration-dependent manner. The mean serum cysteine concentrations in our study, 183 μmol/L, suggest that the protective effects of cysteine on copper-dependent LDL oxidation might prevail over its pro-oxidative effects. This might explain the negative association between cysteine and cardiovascular risk scores. The potential anti-atherogenic effects of cysteine are further supported by recent evidence demonstrating that cysteine reduced calcification of atherosclerotic lesions in apoE-deficient mice and exerted significant anti-inflammatory effects in human coronary endothelial cells [28,29].

The contrasting associations between homocysteine, cysteine and cardiovascular risk are supported by the finding of a positive independent association between homocysteine/cysteine ratios and risk scores. The biotransformation of homocysteine into cysteine is catalyzed by the enzymes cystathionine β-synthase and cystathionine γ-lyase [30]. Therefore, associations between higher homocysteine/cysteine ratios and cardiovascular risk suggest reduced expression and/or activity of these enzymes. Cystathionine β-synthase is the first, rate-limiting, enzyme in the transsulfuration pathway. Congenital cystathionine β-synthase deficiency in humans and knock-out animal models are both typically associated with hyperhomocysteinemia, premature atherosclerosis and vascular disease [31]. Both cystathionine β-synthase and cystathionine γ-lyase are also involved in the synthesis of hydrogen sulphide. The latter is increasingly recognised as having potential anti-atherosclerotic effects in experimental models [32].

Reduced serum glutathione concentrations were also independently associated with higher cardiovascular risk scores in our study. The role of glutathione deficiency in favouring oxidative stress, vascular damage and atherosclerosis is well established [33]. Glutathione deficiency might be secondary to reduced synthesis, increased degradation or a combination of the two processes. In contrast to its synthesis, which occurs within the intracellular compartment through the activity of glutathione synthetase, glutathione degradation into cysteinylglycine by γ-glutamyltranspeptidase occurs in the extracellular space [33]. An increasing body of evidence suggests that γ-glutamyltranspeptidase activity is associated with surrogate markers of vascular disease and predicts adverse cardiovascular outcomes [34,35]. The observation of independent associations between lower glutathione/cysteinylglycine, but not glutamylcysteine/glutathione, ratios and risk scores suggests that increased glutathione degradation, rather than reduced synthesis, plays a major role.

An important finding in our study was the lack of significant associations between taurine concentrations and cardiovascular risk. A number of experimental and human studies have shown that taurine exerts protective effects on vascular homeostasis, including anti-oxidant effects, reduced insulin resistance, improved endothelial function and blood pressure control and reduced serum cholesterol concentrations [36]. However, a recent prospective, nested case–control study assessing the predictive role of serum taurine concentrations on the risk of coronary heart disease has failed to demonstrate any significant effects on the primary end-point [37]. Notably, mean serum taurine concentrations in cases and controls (126.9 vs. 130.1 μmol/L) were higher than those observed in our study (median 62.0 μmol/L, Table 1). Pending further studies on the predictive role of taurine on cardiovascular outcomes the role of this thiol on vascular homeostasis and disease prevention might need some revisiting.

The lack of significant associations between serum CRP concentrations and cardiovascular risk scores in our study is in contrast with the results of several reports demonstrating a significant relationship between serum/plasma CRP concentrations, prevalence of atherosclerosis and incidence of first cardiovascular events [38]. There are at least two potential reasons to explain this finding. First, a substantial proportion (70.9%) of our study participants had low cardiovascular risk (<10%) whereas a very small percentage (6.6%) was at high risk (>15%, Table 1). Previous studies have investigated the relationship between CRP and Framingham coronary heart disease scores in subjects without clinical cardiovascular disease [39]. Although CRP concentrations were significantly correlated with risk scores the relationship between the two variables was fairly flat in patients with low-moderate risk. It is possible that the absence of significant associations between CRP and cardiovascular risk scores in our study is at least partly caused by the low representation of patients at high risk. Interestingly, in their study Albert et al. failed to observe significant correlations between CRP concentrations and several individual components of the Framingham risk score [39]. Our observations are in line with their findings (Table 2). Second, the age range of our population was 59–66 years. There is evidence that the predictive role of CRP is somewhat diminished with advancing age. Several studies have demonstrated that CRP concentrations exert a negligible effect in enhancing cardiovascular risk prediction, over and above traditional risk factors, in older adults [40,41]. In one of these studies homocysteine showed the best predictive power for cardiovascular mortality [41].

A limitation of our study is related to its cross-sectional nature, which does not allow the assessment of cause-effect relationship between transsulfuration thiols and cardiovascular risk. Moreover, the measurement of thiol concentrations from blood does not necessarily reflect their intracellular concentrations. Strength of this study was the combined assessment of different components of the transsulfuration pathway, and their ratios, in regression analysis, adjusting for important clinical confounders.

## Conclusions

Serum concentrations of the thiols homocysteine, cysteine and glutathione are independently associated with cardiovascular risk scores at population level. Analysis of substrate/product ratios suggests that reduced bioconversion of homocysteine into cysteine and increased glutathione degradation might play a pivotal role in such associations. Further studies are required to identify whether abnormal expression and activity of the enzymes cystathionine β-synthase, cystathionine γ-lyase and γ-glutamyltranspeptidase are associated with surrogate cardiovascular markers, adverse cardiovascular outcomes in longitudinal studies and whether their pharmacological and/or non-pharmacological modulation might lead to reduced risk.

## Competing interests

The authors declare that they have no competing interests.

## Authors’ contributions

AAM and MM formulated the initial hypothesis. MM and JA collected the clinical and demographic data. AAM, AZ, CC and MM analysed the biochemical data and interpreted the results. AAM wrote the first draft. All authors critically reviewed the manuscript before its final version. All authors read and approved the final manuscript.

## Authors’ information

Arduino Mangoni conducted this work during a Visiting Professorship at the University of Sassari.
